# 2,7-Dimeth­oxy-1-(2-naphtho­yl)naph­thalene

**DOI:** 10.1107/S1600536812033545

**Published:** 2012-07-28

**Authors:** Takehiro Tsumuki, Atsumi Isogai, Atsushi Nagasawa, Akiko Okamoto, Noriyuki Yonezawa

**Affiliations:** a Department of Organic and Polymer Materials Chemistry, Tokyo University of Agriculture & Technology, 2-24-16 Naka-machi, Koganei, Tokyo 184-8588, Japan

## Abstract

In the title mol­ecule, C_23_H_18_O_3_, the dihedral angle between the two naphthalene ring systems is 80.44 (4)°. The mean plane of the bridging carbonyl C—C(=O)—C group makes a torsion angle of −68.55 (17)° with the naphthalene system of the 2,7-dimeth­oxy­naphthalene unit and a torsion angle of −9.01 (19)° with the naphthalene ring system of the naphthoyl group. In the crystal, a weak C—H⋯O hydrogen bond occurs between the carbonyl O atom and an H atom of the naphthalene ring in the 2,7-dimeth­oxy­naphthalene unit of a symmetry-related mol­ecule.

## Related literature

For electrophilic aromatic aroylation of naphthalene derivatives, see: Okamoto & Yonezawa (2009 ▶); Okamoto *et al.* (2011 ▶). For the structures of closely related compounds, see: Kato *et al.* (2010 ▶); Muto *et al.* (2011 ▶, 2012 ▶); Nakaema *et al.* (2008 ▶); Tsumuki *et al.* (2011 ▶).
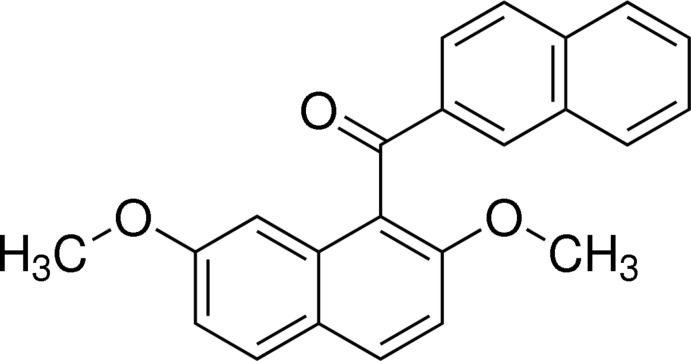


## Experimental

### 

#### Crystal data

C_23_H_18_O_3_
*M* *_r_* = 342.37
Monoclinic, 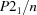

*a* = 11.2483 (3) Å
*b* = 12.2309 (3) Å
*c* = 12.7494 (3) Å
β = 91.936 (1)°
*V* = 1753.01 (7) Å^3^
*Z* = 4
Cu *K*α radiation
μ = 0.68 mm^−1^
*T* = 193 K
0.60 × 0.20 × 0.20 mm

#### Data collection

Rigaku R-AXIS RAPID diffractometer
Absorption correction: numerical (*NUMABS*; Higashi, 1999 ▶) *T*_min_ = 0.685, *T*_max_ = 0.876
27593 measured reflections
3178 independent reflections
2457 reflections with *I* > 2σ(*I*)
*R*_int_ = 0.035

#### Refinement

*R*[*F* ^2^ > 2σ(*F* ^2^)] = 0.043
*wR*(*F* ^2^) = 0.126
*S* = 1.07
3178 reflections
238 parameters
H-atom parameters constrained
Δρ_max_ = 0.20 e Å^−3^
Δρ_min_ = −0.14 e Å^−3^

### 

Data collection: *PROCESS-AUTO* (Rigaku, 1998 ▶); cell refinement: *PROCESS-AUTO*; data reduction: *CrystalStructure* (Rigaku/MSC, 2004 ▶); program(s) used to solve structure: *SIR2004* (Burla *et al.*, 2005 ▶); program(s) used to refine structure: *SHELXL97* (Sheldrick, 2008 ▶); molecular graphics: *ORTEPIII* (Burnett & Johnson, 1996 ▶); software used to prepare material for publication: *SHELXL97*.

## Supplementary Material

Crystal structure: contains datablock(s) I, global. DOI: 10.1107/S1600536812033545/lh5502sup1.cif

Structure factors: contains datablock(s) I. DOI: 10.1107/S1600536812033545/lh5502Isup2.hkl

Supplementary material file. DOI: 10.1107/S1600536812033545/lh5502Isup3.cml

Additional supplementary materials:  crystallographic information; 3D view; checkCIF report

## Figures and Tables

**Table 1:** Hydrogen-bond geometry (Å, °)

*D*—H⋯*A*	*D*—H	H⋯*A*	*D*⋯*A*	*D*—H⋯*A*
C4—H4⋯O1^i^	0.95	2.51	3.2804 (17)	138

Symmetry code: (i) 
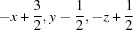
.

**Fig. 1.**
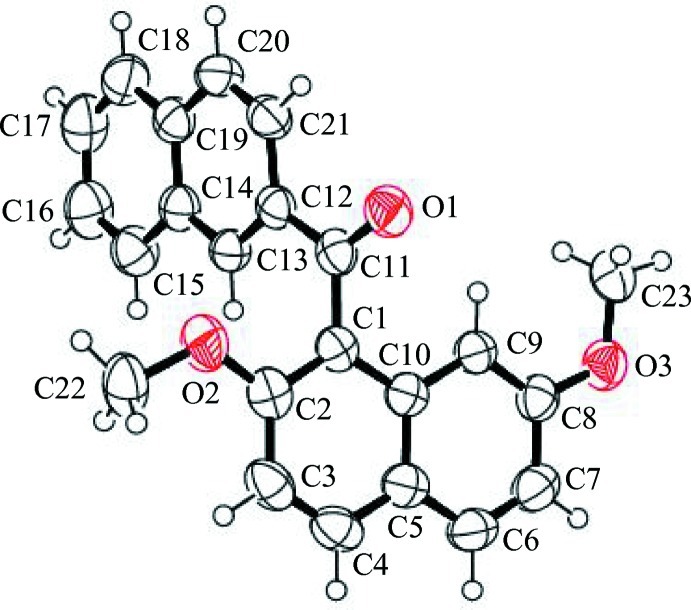
The molecular structure of compound (I), showing displacement ellipsoids drawn at the 50% probability level.

**Fig. 2.**
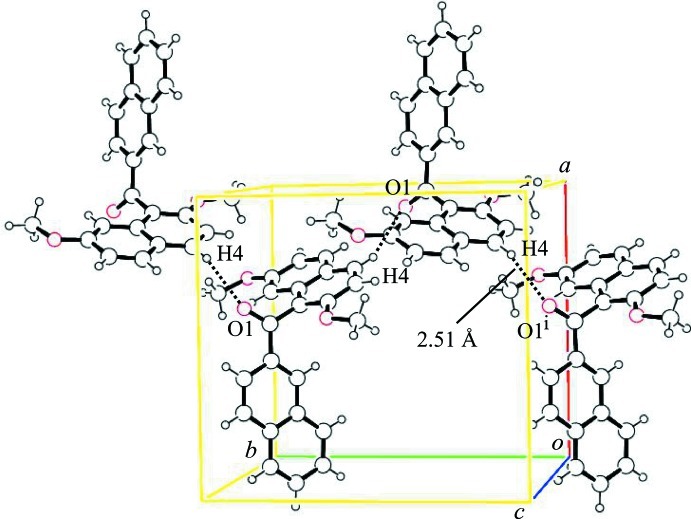
A partial packing diagram of compound (I) with C—H···O interactions shown as dashed lines [symmetry code: (i) -x+2/3, y-1/2, -z+1/2].

## supplementary crystallographic information

### Comment

In the course of our study on electrophilic aromatic aroylation of 2,7-dimethoxynaphthalene, 1,8-diaroylnaphthalene compounds have proven to be formed regioselectively with the aid of suitable acidic mediators (Okamoto & Yonezawa, 2009, Okamoto *et al.*, 2011). Recently, we have reported the crystal structures of several 1,8-diaroylated naphthalene homologues exemplified by 1,8-dibenzoyl-2,7-dimethoxynaphthalene (Nakaema *et al.*, 2008), [2,7-dimethoxy-8-(2,4,6-trimethylbenzoyl)naphthalen-1-yl](2,4,6-trimethylphenyl)methanone (Muto *et al.*, 2012) and [2,7-dimethoxy-8-(2-naphthoyl)naphthalen-1-yl](naphthalen-2-yl)methanone (Tsumuki *et al.*, 2011). The aroyl groups at the 1,8-positions of the naphthalene rings in these compounds are connected almost perpendicularly and oriented in opposite directions.

The crystal structures of 1-monoaroylated naphthalene compounds have essentially the same non-coplanar structure as the 1,8-diaroylated naphthalene compounds, *e.g.*, (2,7-dimethoxynaphthalen-1-yl)(phenyl)methanone (Kato *et al.*, 2010), (2,7-dimethoxynaphthalen-1-yl)(2,4,6-trimethylphenyl)methanone (Muto *et al.*, 2011).

As a part of the course of our continuous study on the molecular structures of these type of homologous molecules, the crystal structure of title compound (I), 1-(2-naphthoyl)-2,7-dimethoxynaphthalene, is discussed in this paper.

The molecular structure of (I) is displayed in Fig. 1. The interplanar angle between the two naphthalene ring systems (C1—C10 and C12—C21) is 80.44 (4)°. The torsion angle between the carbonyl group and the naphthalene ring of 2,7-dimethoxynaphthalene moiety [C10—C1—C11—O1 = -68.55 (17)°] is larger than that between the carbonyl group and naphthalene ring of naphthoyl group [O1—C11—C12—C21 = -9.01 (19)°]. The molecular packing of (I) is mainly stabilized by weak intermolecular hydrogen bonds between the oxygen atom of the carbonyl group and a hydrogen atom of the 2,7-dimethoxynaphthalene unit along *b* axis (Table 1 and Fig. 2).

### Experimental

To a solution of 2-naphthoyl chloride (1.7 g, 8.9 mmol), AlCl_3_ (1.8 g, 13 mmol) and CH_2_Cl_2_ (40 ml), 2,7-dimethoxynaphthalene (1.5 g, 8.1 mmol) was added. The reaction mixture was stirred at 273 K for 6 h, then poured into ice-cold water (40 ml) and the aqueous layer was extracted with CHCl_3_ (20 ml × 3). The combined organic extracts were washed with 2 *M* aqueous NaOH (20 ml × 3) followed by washing with brine (20 ml × 3). The organic layer was dried over anhydrous MgSO_4_. The solvent was removed under reduced pressure to give a cake (83% yield). The crude product was purified by recrystallization from ethanol (36% isolated yield). Single crystals suitable for X-ray diffraction were obtained by repeated crystallization from ethanol.

Spectroscopic data: ^1^H NMR δ (300 MHz, CDCl_3_): 3.69 (3*H*, s), 3.78 (3*H*, s), 6.84 (1*H*, d, J = 2.4 Hz), 7.03 (1*H*, dd, J = 2.4, 9.0 Hz), 7.21 (1*H*, d, J = 9.0 Hz), 7.49 (1*H*, dt, J = 1.2, 7.5 Hz), 7.58 (1*H*, dt, J = 1.2, 7.5 Hz), 7.76 (1*H*, d, J = 9.0), 7.82 (1*H*, d, J = 9.0 Hz), 7.87–7.93 (3*H*, m), 8.07 (1*H*, dd, J = 1.2, 9.0 Hz), 8.24 (1*H*, d, J = 1.2 Hz) p.p.m.; ^13^C NMR δ (75 MHz, CDCl_3_): 55.14, 56.31, 102.07, 110.24, 117.12, 121.88, 124.38, 124.59, 126.52, 127.75, 128.39, 128.50, 129.66, 129.71, 131.00, 131.97, 132.60, 133.16, 135.45, 135.86, 155.02, 155.85, 198.12 p.p.m.; IR (KBr): 1660, 1624, 1511, 1465, 1253 cm^-1^; HRMS (*m/z*): [*M*+H]^+^ calcd. for C_23_H_19_O_3_, 343.1334, found, 343.1310; m.p. = 413.0–414.5 K

### Refinement

All H atoms were found in a difference Fourier map and were subsequently refined as riding atoms, with C—H = 0.95 (aromatic) and 0.98 (methyl) Å, and with *U*_iso_(H) = 1.2*U*_eq_(C).

### Crystal data

**Table:**

C_23_H_18_O_3_	*F*(000) = 720
*M**_r_* = 342.37	*D*_x_ = 1.297 Mg m^−^^3^
Monoclinic, *P*2_1_/*n*	Melting point = 413.0–414.5 K
Hall symbol: -P 2yn	Cu *K*α radiation, λ = 1.54187 Å
*a* = 11.2483 (3) Å	Cell parameters from 19666 reflections
*b* = 12.2309 (3) Å	θ = 3.5–68.2°
*c* = 12.7494 (3) Å	µ = 0.68 mm^−^^1^
β = 91.936 (1)°	*T* = 193 K
*V* = 1753.01 (7) Å^3^	Block, colorless
*Z* = 4	0.60 × 0.20 × 0.20 mm

### Data collection

**Table:**

Rigaku R-AXIS RAPID diffractometer	3178 independent reflections
Radiation source: rotating anode	2457 reflections with *I* > 2σ(*I*)
Graphite monochromator	*R*_int_ = 0.035
Detector resolution: 10.00 pixels mm^-1^	θ_max_ = 68.2°, θ_min_ = 5.0°
ω scans	*h* = −13→13
Absorption correction: numerical (*NUMABS*; Higashi, 1999)	*k* = −14→14
*T*_min_ = 0.685, *T*_max_ = 0.876	*l* = −14→15
27593 measured reflections	

### Refinement

**Table:**

Refinement on *F*^2^	Secondary atom site location: difference Fourier map
Least-squares matrix: full	Hydrogen site location: inferred from neighbouring sites
*R*[*F*^2^ > 2σ(*F*^2^)] = 0.043	H-atom parameters constrained
*wR*(*F*^2^) = 0.126	*w* = 1/[σ^2^(*F*_o_^2^) + (0.0802*P*)^2^] where *P* = (*F*_o_^2^ + 2*F*_c_^2^)/3
*S* = 1.07	(Δ/σ)_max_ < 0.001
3178 reflections	Δρ_max_ = 0.20 e Å^−^^3^
238 parameters	Δρ_min_ = −0.14 e Å^−^^3^
0 restraints	Extinction correction: *SHELXL97* (Sheldrick, 1997), Fc^*^=kFc[1+0.001xFc^2^λ^3^/sin(2θ)]^-1/4^
Primary atom site location: structure-invariant direct methods	Extinction coefficient: 0.0072 (7)

### Special details

**Table:**

Geometry. All e.s.d.'s (except the e.s.d. in the dihedral angle between two l.s. planes) are estimated using the full covariance matrix. The cell e.s.d.'s are taken into account individually in the estimation of e.s.d.'s in distances, angles and torsion angles; correlations between e.s.d.'s in cell parameters are only used when they are defined by crystal symmetry. An approximate (isotropic) treatment of cell e.s.d.'s is used for estimating e.s.d.'s involving l.s. planes.
Refinement. Refinement of *F*^2^ against ALL reflections. The weighted *R*-factor *wR* and goodness of fit *S* are based on *F*^2^, conventional *R*-factors *R* are based on *F*, with *F* set to zero for negative *F*^2^. The threshold expression of *F*^2^ > σ(*F*^2^) is used only for calculating *R*-factors(gt) *etc*. and is not relevant to the choice of reflections for refinement. *R*-factors based on *F*^2^ are statistically about twice as large as those based on *F*, and *R*- factors based on ALL data will be even larger.

### Fractional atomic coordinates and isotropic or equivalent isotropic displacement parameters (Å^2^)

**Table:**

	*x*	*y*	*z*	*U*_iso_*/*U*_eq_	
O1	0.57798 (9)	1.01436 (8)	0.35896 (8)	0.0637 (3)	
O2	0.54637 (10)	0.73226 (8)	0.39846 (9)	0.0686 (3)	
O3	0.65809 (9)	1.09739 (8)	−0.03426 (8)	0.0623 (3)	
C1	0.58205 (11)	0.85111 (10)	0.26051 (11)	0.0469 (3)	
C2	0.59439 (12)	0.74753 (11)	0.30201 (12)	0.0549 (4)	
C3	0.65526 (13)	0.66529 (12)	0.24865 (14)	0.0614 (4)	
H3	0.6652	0.5949	0.2791	0.074*	
C4	0.70002 (12)	0.68740 (12)	0.15252 (14)	0.0600 (4)	
H4	0.7400	0.6312	0.1163	0.072*	
C5	0.68833 (11)	0.79121 (11)	0.10605 (12)	0.0521 (4)	
C6	0.73236 (13)	0.81473 (13)	0.00565 (13)	0.0599 (4)	
H6	0.7718	0.7590	−0.0317	0.072*	
C7	0.71939 (13)	0.91483 (13)	−0.03809 (13)	0.0617 (4)	
H7	0.7483	0.9284	−0.1060	0.074*	
C8	0.66278 (12)	0.99956 (11)	0.01716 (12)	0.0522 (4)	
C9	0.61945 (11)	0.98103 (11)	0.11472 (11)	0.0469 (3)	
H9	0.5824	1.0387	0.1514	0.056*	
C10	0.62990 (11)	0.87576 (10)	0.16099 (11)	0.0461 (3)	
C11	0.52073 (12)	0.93829 (10)	0.32143 (10)	0.0475 (3)	
C12	0.38946 (11)	0.93372 (10)	0.33175 (10)	0.0451 (3)	
C13	0.32116 (12)	0.86080 (10)	0.27471 (10)	0.0474 (3)	
H13	0.3589	0.8086	0.2318	0.057*	
C14	0.19544 (12)	0.86165 (11)	0.27837 (11)	0.0501 (4)	
C15	0.12365 (14)	0.78729 (13)	0.21902 (13)	0.0640 (4)	
H15	0.1602	0.7347	0.1757	0.077*	
C16	0.00259 (15)	0.79033 (15)	0.22335 (15)	0.0744 (5)	
H16	−0.0445	0.7398	0.1835	0.089*	
C17	−0.05224 (15)	0.86780 (15)	0.28653 (15)	0.0754 (5)	
H17	−0.1366	0.8704	0.2882	0.091*	
C18	0.01400 (14)	0.93934 (13)	0.34559 (15)	0.0689 (5)	
H18	−0.0246	0.9908	0.3887	0.083*	
C19	0.14025 (12)	0.93802 (11)	0.34363 (12)	0.0539 (4)	
C20	0.21309 (14)	1.01101 (11)	0.40415 (12)	0.0596 (4)	
H20	0.1771	1.0618	0.4497	0.071*	
C21	0.33359 (13)	1.00932 (10)	0.39785 (11)	0.0539 (4)	
H21	0.3806	1.0594	0.4383	0.065*	
C22	0.53909 (15)	0.62372 (14)	0.43784 (16)	0.0768 (5)	
H22A	0.4942	0.6238	0.5024	0.092*	
H22B	0.6194	0.5956	0.4529	0.092*	
H22C	0.4986	0.5771	0.3853	0.092*	
C23	0.60777 (14)	1.18752 (12)	0.01878 (13)	0.0630 (4)	
H23A	0.6071	1.2518	−0.0271	0.076*	
H23B	0.6554	1.2032	0.0828	0.076*	
H23C	0.5262	1.1697	0.0373	0.076*	

### Atomic displacement parameters (Å^2^)

**Table:**

	*U*^11^	*U*^22^	*U*^33^	*U*^12^	*U*^13^	*U*^23^
O1	0.0661 (7)	0.0625 (6)	0.0624 (7)	−0.0174 (5)	0.0026 (5)	−0.0102 (5)
O2	0.0813 (7)	0.0587 (6)	0.0657 (8)	0.0023 (5)	0.0033 (6)	0.0205 (5)
O3	0.0723 (7)	0.0621 (6)	0.0529 (7)	−0.0069 (5)	0.0077 (5)	0.0067 (5)
C1	0.0420 (7)	0.0463 (7)	0.0520 (8)	−0.0019 (5)	−0.0043 (6)	0.0017 (6)
C2	0.0496 (8)	0.0526 (8)	0.0618 (10)	−0.0033 (6)	−0.0071 (7)	0.0077 (7)
C3	0.0512 (8)	0.0457 (8)	0.0864 (12)	0.0036 (6)	−0.0109 (8)	0.0049 (8)
C4	0.0464 (8)	0.0503 (8)	0.0826 (12)	0.0051 (6)	−0.0065 (8)	−0.0095 (8)
C5	0.0396 (7)	0.0525 (8)	0.0638 (10)	−0.0007 (6)	−0.0039 (6)	−0.0090 (7)
C6	0.0500 (8)	0.0650 (9)	0.0650 (11)	−0.0008 (6)	0.0069 (7)	−0.0196 (8)
C7	0.0572 (9)	0.0729 (10)	0.0554 (10)	−0.0071 (7)	0.0090 (7)	−0.0105 (8)
C8	0.0488 (7)	0.0554 (8)	0.0524 (9)	−0.0073 (6)	0.0010 (6)	−0.0007 (7)
C9	0.0446 (7)	0.0483 (7)	0.0478 (8)	−0.0017 (5)	0.0007 (6)	−0.0023 (6)
C10	0.0376 (6)	0.0486 (7)	0.0516 (8)	−0.0021 (5)	−0.0038 (6)	−0.0031 (6)
C11	0.0561 (8)	0.0465 (7)	0.0396 (8)	−0.0056 (6)	−0.0024 (6)	0.0046 (6)
C12	0.0531 (7)	0.0418 (7)	0.0405 (8)	−0.0005 (5)	0.0011 (6)	0.0063 (6)
C13	0.0540 (8)	0.0441 (7)	0.0444 (8)	0.0008 (6)	0.0038 (6)	0.0027 (6)
C14	0.0515 (8)	0.0496 (7)	0.0491 (8)	−0.0004 (6)	0.0002 (6)	0.0092 (6)
C15	0.0572 (9)	0.0636 (9)	0.0710 (11)	−0.0059 (7)	−0.0031 (8)	−0.0011 (8)
C16	0.0567 (9)	0.0765 (11)	0.0892 (13)	−0.0107 (8)	−0.0086 (9)	0.0097 (10)
C17	0.0506 (9)	0.0790 (11)	0.0964 (14)	0.0009 (8)	−0.0017 (9)	0.0259 (11)
C18	0.0591 (9)	0.0680 (10)	0.0802 (12)	0.0134 (8)	0.0125 (8)	0.0169 (9)
C19	0.0561 (8)	0.0493 (8)	0.0566 (9)	0.0057 (6)	0.0055 (7)	0.0125 (7)
C20	0.0692 (10)	0.0500 (8)	0.0602 (10)	0.0099 (7)	0.0124 (8)	−0.0014 (7)
C21	0.0672 (9)	0.0435 (7)	0.0511 (9)	−0.0003 (6)	0.0027 (7)	0.0006 (6)
C22	0.0661 (10)	0.0666 (10)	0.0976 (14)	−0.0009 (8)	0.0030 (9)	0.0336 (10)
C23	0.0707 (10)	0.0544 (8)	0.0638 (10)	−0.0056 (7)	−0.0012 (8)	0.0080 (7)

### Geometric parameters (Å, º)

**Table:**

O1—C11	1.2200 (15)	C12—C21	1.4128 (18)
O2—C2	1.3724 (18)	C13—C14	1.4165 (19)
O2—C22	1.4227 (18)	C13—H13	0.9500
O3—C8	1.3645 (16)	C14—C19	1.4090 (19)
O3—C23	1.4209 (18)	C14—C15	1.418 (2)
C1—C2	1.3781 (18)	C15—C16	1.365 (2)
C1—C10	1.4271 (19)	C15—H15	0.9500
C1—C11	1.5008 (18)	C16—C17	1.400 (2)
C2—C3	1.405 (2)	C16—H16	0.9500
C3—C4	1.368 (2)	C17—C18	1.360 (2)
C3—H3	0.9500	C17—H17	0.9500
C4—C5	1.405 (2)	C18—C19	1.421 (2)
C4—H4	0.9500	C18—H18	0.9500
C5—C6	1.418 (2)	C19—C20	1.422 (2)
C5—C10	1.4224 (18)	C20—C21	1.361 (2)
C6—C7	1.351 (2)	C20—H20	0.9500
C6—H6	0.9500	C21—H21	0.9500
C7—C8	1.416 (2)	C22—H22A	0.9800
C7—H7	0.9500	C22—H22B	0.9800
C8—C9	1.3699 (19)	C22—H22C	0.9800
C9—C10	1.4195 (18)	C23—H23A	0.9800
C9—H9	0.9500	C23—H23B	0.9800
C11—C12	1.4879 (17)	C23—H23C	0.9800
C12—C13	1.3699 (18)		
			
C2—O2—C22	118.18 (13)	C12—C13—H13	119.3
C8—O3—C23	117.46 (11)	C14—C13—H13	119.3
C2—C1—C10	119.96 (13)	C19—C14—C13	118.98 (13)
C2—C1—C11	119.77 (13)	C19—C14—C15	119.08 (14)
C10—C1—C11	120.25 (11)	C13—C14—C15	121.93 (13)
O2—C2—C1	115.56 (13)	C16—C15—C14	120.79 (16)
O2—C2—C3	123.32 (13)	C16—C15—H15	119.6
C1—C2—C3	121.10 (14)	C14—C15—H15	119.6
C4—C3—C2	119.50 (14)	C15—C16—C17	120.06 (16)
C4—C3—H3	120.2	C15—C16—H16	120.0
C2—C3—H3	120.2	C17—C16—H16	120.0
C3—C4—C5	121.62 (14)	C18—C17—C16	120.67 (16)
C3—C4—H4	119.2	C18—C17—H17	119.7
C5—C4—H4	119.2	C16—C17—H17	119.7
C4—C5—C6	122.24 (13)	C17—C18—C19	120.80 (16)
C4—C5—C10	119.17 (14)	C17—C18—H18	119.6
C6—C5—C10	118.59 (13)	C19—C18—H18	119.6
C7—C6—C5	121.36 (14)	C14—C19—C18	118.57 (14)
C7—C6—H6	119.3	C14—C19—C20	118.65 (13)
C5—C6—H6	119.3	C18—C19—C20	122.77 (14)
C6—C7—C8	120.12 (15)	C21—C20—C19	121.03 (14)
C6—C7—H7	119.9	C21—C20—H20	119.5
C8—C7—H7	119.9	C19—C20—H20	119.5
O3—C8—C9	124.89 (13)	C20—C21—C12	120.61 (14)
O3—C8—C7	114.42 (13)	C20—C21—H21	119.7
C9—C8—C7	120.68 (13)	C12—C21—H21	119.7
C8—C9—C10	120.02 (12)	O2—C22—H22A	109.5
C8—C9—H9	120.0	O2—C22—H22B	109.5
C10—C9—H9	120.0	H22A—C22—H22B	109.5
C9—C10—C5	119.21 (13)	O2—C22—H22C	109.5
C9—C10—C1	122.19 (12)	H22A—C22—H22C	109.5
C5—C10—C1	118.60 (12)	H22B—C22—H22C	109.5
O1—C11—C12	120.41 (12)	O3—C23—H23A	109.5
O1—C11—C1	119.94 (12)	O3—C23—H23B	109.5
C12—C11—C1	119.59 (11)	H23A—C23—H23B	109.5
C13—C12—C21	119.34 (12)	O3—C23—H23C	109.5
C13—C12—C11	121.20 (12)	H23A—C23—H23C	109.5
C21—C12—C11	119.40 (12)	H23B—C23—H23C	109.5
C12—C13—C14	121.36 (12)		
			
C22—O2—C2—C1	170.08 (12)	C11—C1—C10—C5	179.43 (11)
C22—O2—C2—C3	−11.5 (2)	C2—C1—C11—O1	110.03 (15)
C10—C1—C2—O2	179.41 (11)	C10—C1—C11—O1	−68.54 (17)
C11—C1—C2—O2	0.83 (18)	C2—C1—C11—C12	−72.56 (16)
C10—C1—C2—C3	1.0 (2)	C10—C1—C11—C12	108.87 (14)
C11—C1—C2—C3	−177.59 (12)	O1—C11—C12—C13	167.99 (12)
O2—C2—C3—C4	179.77 (13)	C1—C11—C12—C13	−9.41 (18)
C1—C2—C3—C4	−1.9 (2)	O1—C11—C12—C21	−9.02 (18)
C2—C3—C4—C5	1.0 (2)	C1—C11—C12—C21	173.58 (12)
C3—C4—C5—C6	−178.83 (13)	C21—C12—C13—C14	1.80 (19)
C3—C4—C5—C10	0.8 (2)	C11—C12—C13—C14	−175.22 (11)
C4—C5—C6—C7	179.45 (13)	C12—C13—C14—C19	−1.14 (19)
C10—C5—C6—C7	−0.2 (2)	C12—C13—C14—C15	179.42 (13)
C5—C6—C7—C8	1.2 (2)	C19—C14—C15—C16	1.0 (2)
C23—O3—C8—C9	2.04 (19)	C13—C14—C15—C16	−179.52 (14)
C23—O3—C8—C7	−176.96 (12)	C14—C15—C16—C17	0.3 (3)
C6—C7—C8—O3	178.34 (13)	C15—C16—C17—C18	−1.2 (3)
C6—C7—C8—C9	−0.7 (2)	C16—C17—C18—C19	0.7 (2)
O3—C8—C9—C10	−179.62 (12)	C13—C14—C19—C18	179.07 (13)
C7—C8—C9—C10	−0.68 (19)	C15—C14—C19—C18	−1.5 (2)
C8—C9—C10—C5	1.59 (18)	C13—C14—C19—C20	−0.49 (19)
C8—C9—C10—C1	−177.45 (11)	C15—C14—C19—C20	178.97 (13)
C4—C5—C10—C9	179.17 (12)	C17—C18—C19—C14	0.6 (2)
C6—C5—C10—C9	−1.15 (18)	C17—C18—C19—C20	−179.86 (14)
C4—C5—C10—C1	−1.76 (18)	C14—C19—C20—C21	1.5 (2)
C6—C5—C10—C1	177.93 (12)	C18—C19—C20—C21	−178.07 (14)
C2—C1—C10—C9	179.90 (12)	C19—C20—C21—C12	−0.8 (2)
C11—C1—C10—C9	−1.53 (19)	C13—C12—C21—C20	−0.8 (2)
C2—C1—C10—C5	0.86 (18)	C11—C12—C21—C20	176.26 (13)

### Hydrogen-bond geometry (Å, º)

**Table:**

*D*—H···*A*	*D*—H	H···*A*	*D*···*A*	*D*—H···*A*
C4—H4···O1^i^	0.95	2.51	3.2804 (17)	138

Symmetry code: (i) −*x*+3/2, *y*−1/2, −*z*+1/2.
